# Human Umbilical Cord Mesenchymal Stem Cells Therapy in Cyclophosphamide-Induced Premature Ovarian Failure Rat Model

**DOI:** 10.1155/2016/2517514

**Published:** 2016-03-07

**Authors:** Dan Song, Yun Zhong, Chunfeng Qian, Qinyan Zou, Jian Ou, Yichao Shi, Liang Gao, Gaigai Wang, Zhenxing Liu, Haibo Li, Hailei Ding, Huihua Wu, Fuxin Wang, Jing Wang, Hong Li

**Affiliations:** ^1^Center for Reproduction and Genetics, Suzhou Municipal Hospital, Nanjing Medical University Affiliated Suzhou Hospital, Suzhou, Jiangsu 215002, China; ^2^Jiangsu Beike Biotechnology Co., Ltd., Jiangsu 215002, China; ^3^Experimental Animal Center, Soochow University, Suzhou, Jiangsu 215006, China

## Abstract

Premature ovarian failure (POF) is one of the most common causes of infertility in women. In our present study, we established cyclophosphamide- (CTX-) induced POF rat model and elucidated its effect on ovarian function. We detected the serum estrogen, follicle stimulating hormone, and anti-Müllerian hormone of mice models by ELISA and evaluated their folliculogenesis by histopathology examination. Our study revealed that CTX administration could severely disturb hormone secretion and influence folliculogenesis in rat. This study also detected ovarian cells apoptosis by deoxy-UTP-digoxigenin nick end labeling (TUNEL) and demonstrated marked ovarian cells apoptosis in rat models following CTX administration. In order to explore the potential of human umbilical cord mesenchymal stem cells (UCMSCs) in POF treatment, the above indexes were used to evaluate ovarian function. We found that human UCMSCs transplantation recovered disturbed hormone secretion and folliculogenesis in POF rat, in addition to reduced ovarian cell apoptosis. We also tracked transplanted UCMSCs in ovaries by fluorescence* in situ* hybridization (FISH). The results manifested that the transplanted human UCMSCs could reside in ovarian tissues and could survive for a comparatively long time without obvious proliferation. Our present study provides new insights into the great clinical potential of human UCMSCs in POF treatment.

## 1. Introduction 

Premature ovarian failure (POF) is a heterogeneous disorder, which is defined as the cessation of ovarian function, along with elevated follicle stimulating hormone (FSH) and decreased estrogen (E2) levels in women below 40 years [1]. Ovarian atrophy, reduced follicle stores, menstrual irregularities, and ovarian dysfunction are the most important characteristics of POF. It has become an important cause of infertility. The etiology of POF is complex, which is often caused by genetic defects, autoimmunity, and toxics [2–4]. Nowadays, with the increasing cancer incidence among young women, long term exposure to gonadotoxicity chemotherapy is becoming a major cause of POF.

Cyclophosphamide (CTX), an alkylating agent, has been found to have a deleterious effect on female reproductive organs, especially on ovary [5]. The reversibility of the damage depends on patient's age, exposure degree, and ovarian reserve. The attempts to preserve or restore patients' fertility before or after oncologic therapy have always been a concern.

Gonadotropin and steroid hormone are the key regulators of folliculogenesis in all mammalian species [6]. Disordered endocrine system regulation affects the process of follicular development, follicular storage, and menopausal onset and leads to ovarian pathological conditions, such as POF [7]. Studies have shown that the reduced number of primordial and early antral follicles could reduce serum anti-Müllerian hormone (AMH) level, which reflects follicular storage and is a reliable predictor of POF other than FSH and E2 [8, 9].

Stem cell transplantation has become a promising tool in rescuing damaged ovarian function. Its therapeutic potential has opened up a new way for preserving or recovering the damaged fertility of women receiving chemotherapy [10]. The application of stem cells transplantation is often associated with controversies, such as safety, source, and their poor survival time* in vivo*. Umbilical cord is a rich source of mesenchymal stem cells [11] and the umbilical cord mesenchymal stem cells (UCMSCs) are multipotent, nonhaematopoietic progenitor cells, which can differentiate into multiple cell lineages [12]. The phenotype and characteristic of UCMSCs, as identified by many studies, have many advantages over other MSCs [13–15]. Although its exact protective role in the injured tissues is still obscure, more and more studies have implied that it could inhibit stromal cell apoptosis by secreting growth factors [16–18].

In our current study, we established chemotherapy induced POF rat models, which represent a reliable and physiological model for studying the role of CTX in murine ovarian function. We investigated the potential therapeutic efficiency of human male-derived UCMSCs in rescuing the POF rats. Our study helps point out the potential of UCMSCs in POF treatment and the involved mechanism. We have exhibited the study design in a table (supplementary table 1 in Supplementary Material available online at http://dx.doi.org/10.1155/2016/2517514).

## 2. Materials and Methods

### 2.1. Experimental Animals

Eight-week-old female specific pathogen-free- (SPF-) grade Wistar rats, weighing 180–200 g, were used in our study. The rats were purchased from Soochow University (Suzhou, China). All the animals were housed in animal facility of Soochow University and were fed a standard pellet diet with free access to water. All the experimental procedures were conducted in accordance with the Institutional Animal Care and Use Committee at Soochow University.

### 2.2. Animal Model Establishment

To establish chemotherapy induced POF rat model, rats were randomly divided into two groups: control group and POF group. The POF group rats (*n* = 20) were intraperitoneally injected with 200 mg/kg of CTX (Endoxan, Shionogi & Co., Osaka, Japan) on the first day and then 8 mg/kg/day for the 15 consecutive days. For the control group, we set the blank control group (*n* = 10) without any treatment and negative control group (*n* = 10), which was injected with 0.9% saline instead of CTX.

### 2.3. Human UCMSCs Isolation and Phenotype Determination

UCMSCs were obtained from Beike Biotech Co., Ltd., Jiangsu province. The preparation was as follows: Full-term umbilical cord (UC) was obtained after UC blood removal from newborn boy of normal deliveries after obtaining the informed consent. The isolated UC was manually dissected into 1-2 mm^3^ sections and incubated with 0.075% collagenase type II for 30 min and then 0.125% trypsin for 30 min with gentle agitation at 37°C. UCs were plated at a density of 1 × 10^6^/cm^2^ in cell culture dish containing L-DMEM, supplemented with 5% fetal bovine serum and 1% penicillin/streptomycin at 37°C in a humidified atmosphere of 5% CO_2_. After three days, the nonadherent cells were removed. The first colonies of UCMSCs were obtained after changing the medium several times. Passage 2 cells were harvested with 0.25% trypsin-EDTA and were disaggregated into single cell for further analysis and cells transplantation.

Flow cytometry was used to characterize the human UCMSCs. The following monoclonal antibodies (mAbs) were used: phycoerythrin (PE) conjugated anti-human mAbs against CD29/CD73/CD34 (CD29/CD73: BD Pharmingen, USA; CD34: BD eBioscience, USA); fluorescein isothiocyanate (FITC) conjugated anti-human mAbs against CD90/CD45/CD14 (CD90: BioLegend, USA; CD45/CD14: BD eBioscience, USA); allophycocyanin (APC) conjugated anti-human mAbs against CD105/CD79a (CD105: BD eBioscience, USA; CD79a: BD Pharmingen, USA); and peridinin-chlorophyll protein (perCP) complex conjugated anti-human mAbs against HLA-DR (BD eBioscience, USA).

The harvested UCMSCs were washed twice with FBS-containing PBS and were separately incubated with the above mAbs or mouse IgG isotype control for 30 min at 4°C. Extra mAbs were removed by washing twice with PBS. The cells were resuspended in 0.5 mL PBS to obtain a final concentration of 2 × 10^5^ cells and were analyzed by FACSCalibur Flow Cytometer (BD FACSCalibur).

### 2.4. Stem Cell Transplantation

In order to perform the UCMSCs transplantation, we established the POF rat model in the very same way as previous one. Two weeks after CTX administration, rats were divided into four groups. Group I included rats without any treatment; group II included POF rats model without UCMSCs transplantation; group III included POF rats model injected with male UCMSCs by tail intravenously; and group IV included POF rats model transplanted with UCMSCs* in situ*, with eight rats for each group. Prior to the UCMSCs transplantation, the rats were anesthetized by intraperitoneal injection of ketamine (45 mg/kg body weight). UCMSCs (passage 2 cells) were stored at 4°C in PBS for less than 30 min before transplantation. For tail intravenous trans group, POF rats were transplanted with UCMSCs (100 *μ*L, at a concentration of 1 × 10^6^/mL) intravenously with a microinjector. For* in situ* trans group, POF rats were transplanted directly through each of the bilateral ovaries with UCMSCs (25 *μ*L, at a concentration of 2 × 10^6^/mL) with a microinjector [19, 20].

### 2.5. Hormone Assay

To detect the levels of serum E2, FSH, and AMH, blood samples were obtained from rats models by retroorbital puncture under anesthesia. Samples were incubated at room temperature for 1 h, and supernatant was collected following centrifugation at 4000 r/min for 10 min. The concentration of the hormone was determined by ELISA kits (Yison Bio, Shanghai, China).

According to the kit instructions, rat E2, FSH, and AMH standards were diluted by standard diluent at the final concentration of 0, 5.0, 10, 20, 40, and 80 ng/L, 0, 1.5, 3, 6, 12, and 24 IU/L, and 12.5, 25, 50, 100, and 200 pg/mL, respectively. Fifty microlitres of standard samples was added to the precoated microtest wells and 10 *μ*L plasma was added to 40 *μ*L sample diluent. The whole setting was incubated for 30 min at 37°C. After washing for five times, 50 *μ*L HRP-conjugated reagent was added and the setting was incubated for another 30 min at 37°C. After washing five times, tetramethyl benzidine (TMB) substrate was added to the well, and OD values of the samples were determined by TECAN infinite F80 at a wavelength of 450 nm. According to the standard curve, the concentration of the hormones was determined.

### 2.6. Ovarian Follicle Counting and Morphological Analysis Using Hematoxylin and Eosin Staining

Rats were killed and ovaries were collected at different time intervals following the CTX administration. Ovary tissues were fixed in 10% paraformaldehyde for at least 24 hours. The ovaries were dehydrated and embedded in paraffin and were serially sectioned at 5 *μ*m depth; the sections were stained with hematoxylin and eosin (H&E) for histopathology. The ovarian histological examination was performed using light microscopy (OLYMPUS, Japan). The follicles were detected and classified as primordial, primary, secondary, and early antral follicles, according to the previous description and definition [21]. Follicles containing an oocyte, with a clearly visible nucleus, were counted only.

### 2.7. Apoptosis Assay

In order to detect the cell apoptosis in rat ovaries,* In Situ* Cell Death Detection Kit (KeyGEN BioTech) was used to detect the fragmented DNA histochemically by TUNEL. Fluorescein-labeled nucleotides were incorporated* in situ* onto 3′ ends of DNA strand breaks of the apoptotic cells. According to the instruction, the paraffin sections were dewaxed at 60°C for 60 min and rehydrated with ethanol (100%, 90%, 80%, and 75%) in step by step manner. The slides were washed thrice with PBS for 5 min and incubated with proteinase K at 37°C for 30 min. Fifty-microlitre TdT enzyme reaction mixture was added to the samples, and the whole setting was incubated for 30 min at 37°C in a humidified atmosphere in the dark and washed thrice in PBS for 5 min. Streptavidin-fluorescein reagent was added to the sections and incubated at 37°C in a humidified atmosphere for 30 min in the dark. After washing thrice with PBS, the cell nucleus was dyed with DAPI for 10 min. Apoptotic cells in the ovary were stained green. The sections were observed with fluorescence microscopy (ZEISS Imager A1, Germany).

### 2.8. Stem Cell-Tracking

To determine the location of the transplanted UCMSCs and their fate in ovarian tissues, FISH was performed. Rats were killed at six weeks and eight weeks after UCMSCs transplantation. Ovaries were collected and were fixed in 10% paraformaldehyde for at least 24 hours. The ovaries were sectioned into paraffin with a depth of 5 *μ*m. PathVysion Probe Kit Vysis LSI SRY_Spectrum Orange/CEPX Spectrum Green (Abbott Laboratories, Abbott Park) was used to mark human UCMSCs in ovarian tissue. According to the instruction, the paraffin sections were dewaxed at 60°C for 6 hours and were further dewaxed by treatment of dimethylbenzene three times for 10 min and rehydrated three times with ethanol 100% for 5 min. The sections were denatured with Pretreatment Reagent Kit and were incubated in 80°C for 20 min and were washed with ddH_2_O. The sections were incubated in pepsin at 37°C for 15 min and washed with ddH_2_O. Sections were dehydrated in ethanol (70%, 85%, and 100%) separately for 1 min. Probe preparation: the sections were denatured at 73°C for 10 min and were incubated at 37°C for 7 min. Sections were hybridized with probe at 37°C for 16 h. Following this, the sections were washed in buffer (2x SSC/0.3% NP-40) for 3 min. Counterstaining was carried out with 4,6-diamidino-2-phenylindole (DAPI) for 10 min in the dark. All the sections were visualized using fluorescence microscopy (OLYMPUS, BX53). The location of the UCMSCs was determined using orange (SRY/Y chromosome) and green (CEP/X chromosome) signals.

### 2.9. Statistical Analyses

The follicle numbers were expressed as mean ± standard deviation (SD) and were analyzed by Student's *t*-test and one-way ANOVA. Plasma hormone levels at different time point in each group were analyzed by repeated measures ANOVA, and multiple comparisons of different groups were also conducted. *p* value of < 0.05 was considered to be statistically significant for all of the involved groups, and all analyses were conducted with software of SPSS 16.0.

## 3. Results

### 3.1. Isolation and Characterization of Human UCMSCs

**Figure 1:**
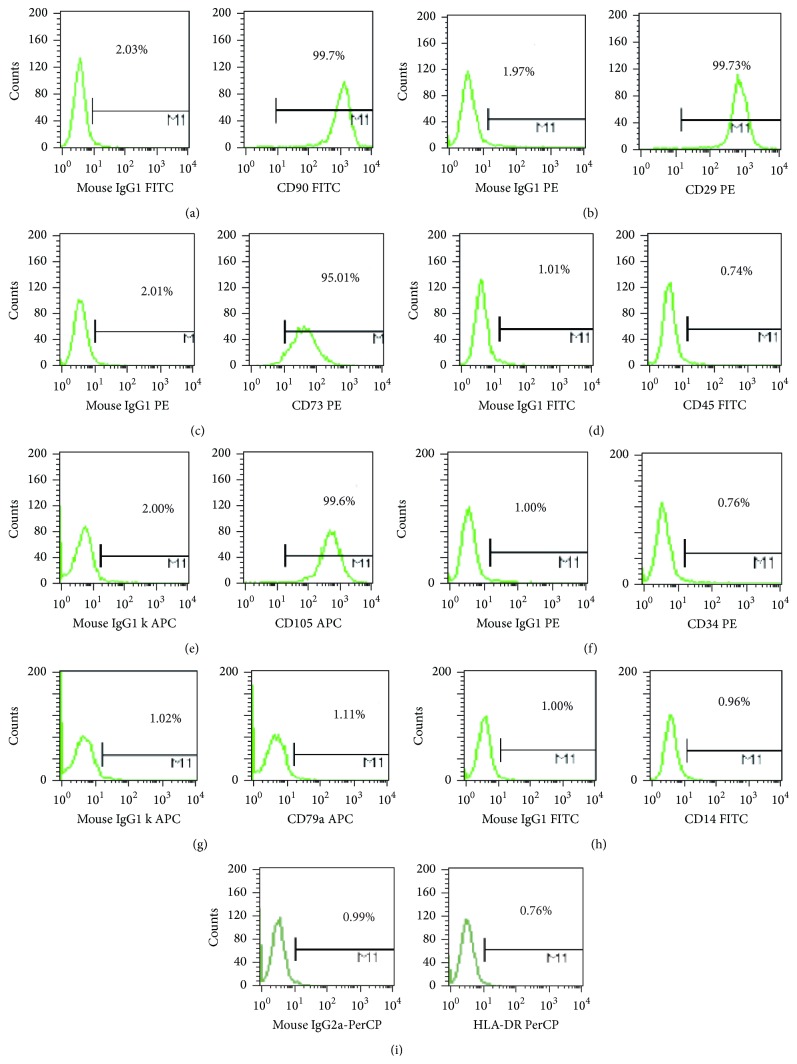
Flow cytometry analysis of human UCMSCs. Flow cytometry analysis of human UCMSCs showed their expression for CD90/CD29/CD73/CD45/CD105/CD34/CD79a/CD14/HLA-DR.

The surface molecular expression of the enriched human UCMSCs was detected using flow cytometry following 2 passages. The results indicated a high expression of CD90/CD29/CD73/CD105 (>95%) for the isolated human UCMSCs (Figures 1(a), 1(b), 1(c), and 1(e)). FACS analysis also reported very low expression levels of CD45/CD34/CD79a/CD14/HLA-DR (<2%) (Figures 1(d), 1(f), 1(g), 1(h), and 1(i)).

### 3.2. Effect of CTX on Ovarian Morphology

**Figure 2:**
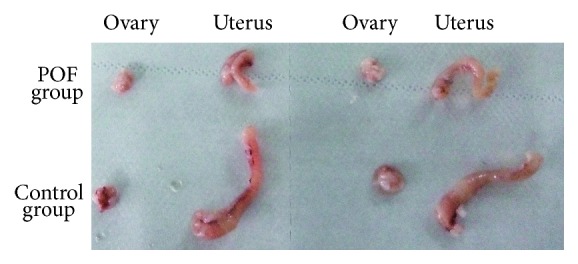
Photographs of ovaries removed from CTX treated groups and control group.

All the animals were weighed before and after the CTX treatment and no significant difference was observed between them. However, although not quantitatively measured, the reduction in the size of reproductive organs (ovaries and uterus) was observed after the CTX treatment (Figure 2). Comparing with the control group, the ovaries and uterus of POF rats were atrophied after two weeks of CTX administration.

### 3.3. Chemotherapy Induced Serum Hormone Level Changes of POF Model

**Figure 3:**
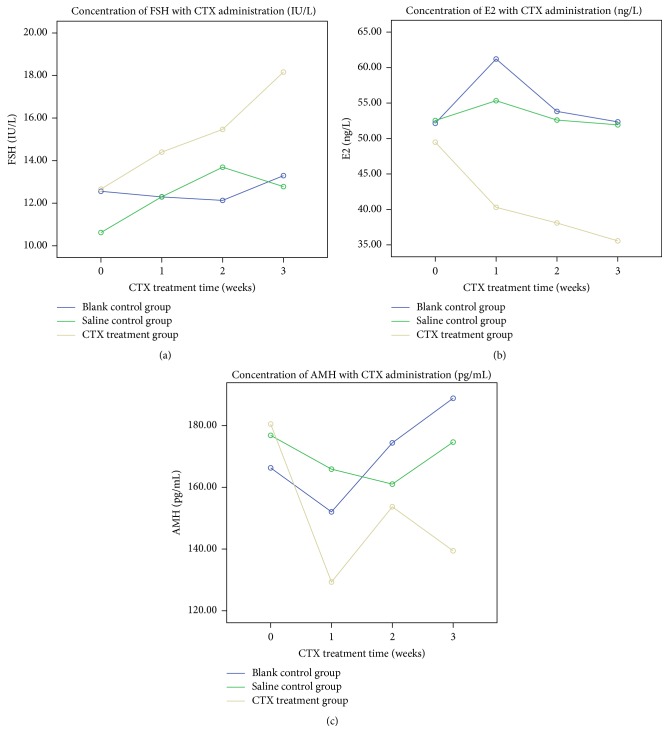
The trend for average level of FSH, AMH, and E2 in each group (blank control group, saline treatment group, and CTX treatment group) during three weeks after CTX treatment.

Blood was obtained from the same four rats of each group after CTX administration for one week, two weeks, and three weeks, respectively. The serum levels of FSH, E2, and AMH were all significantly different among these three groups (blank control group, saline treatment group, and CTX treatment group) (*p* = 0.001, *p* = 0.032, and *p* < 0.0001, resp.). The serum FSH level in CTX treatment group was significantly increased comparing with that in blank control group (*p* = 0.001) (Figure 3(a)). The serum E2 level in CTX treatment group was significantly decreased comparing with that in blank control group (*p* < 0.0001) (Figure 3(b)). AMH level in CTX treatment group was significantly decreased comparing with that in blank control group (*p* = 0.019) (Figure 3(c)). The level of FSH and AMH at different observed times was significantly different (*p* < 0.0001, *p* < 0.0001, resp.), while E2 level was not significantly different at different observed times (*p* = 0.227). Considering that injection process would be an interference factor during CTX administration, we set saline treatment group. According to our study, there was no significant difference in the hormones level between blank control group and saline treatment group (*p* > 0.05); thus the saline treatment group was not included in the following experiments. The data has been presented as Table 1.

### 3.4. Effect of CTX on Follicle Development

Four weeks after CTX administration, three rats from each group were sacrificed for ovarian pathology analysis. In order to detect the effect of CTX administration on the total number of follicles in every stage, we randomly selected five slices of every ovary and classified and counted the follicles on the entire field of these five slices. We separately added the number of follicles in the same stage of these five slices together. Mean of the number of every stage's follicles for two ovaries from one rat was calculated and used for further analysis. We separately calculated the mean numbers of follicles in every stage for three rats per group and perform statistical comparisons.

**Figure 4:**
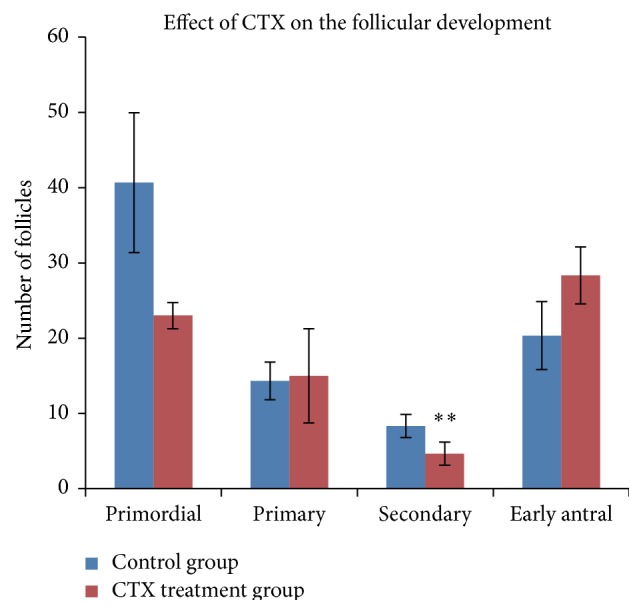
Effect of CTX on the follicles development. Ovarian H&E staining pathological sections were from control group and CTX treatment group. Follicles were counted and classified. Data were means ± SD of counts of different stages follicles (*n* = 3) (primordial, primary, and early antral follicles of POF group versus control group: *p* > 0.05, secondary follicles of POF group versus control group: *p* = 0.004). ^*∗∗*^ *p* < 0.01.

Follicles of every stage were classified according to the following criteria: primordial follicles were identified as follicles with oocytes surrounded by less differentiated squamous granulosa cells (GCs); primary follicles were identified as those with oocytes surrounded by a single layer of cuboidal GCs; secondary follicles were identified as those with oocytes surrounded by two to four complete GC layers; and early antral follicles were identified as those with oocytes surrounded by five or more complete GC layers, with or without a visible antrum. Our results indicated that the number of secondary follicles was significantly decreased in the CTX treatment group (*p* = 0.004), while no significant difference was observed in the number of follicles in other stages between CTX group and control group (Figure 4). The data has been presented in Table 2.

### 3.5. UCMSCs Transplantation Improved Hormone Secretion in POF Rats

**Figure 5:**
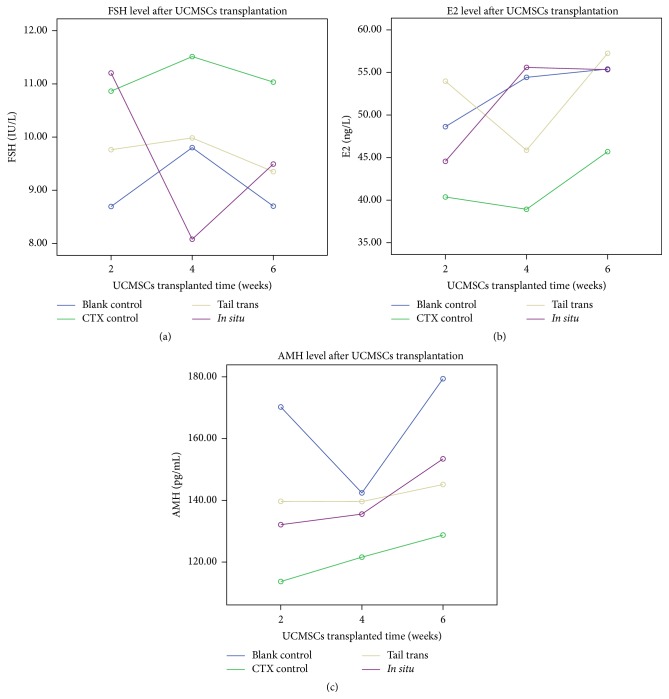
The trend for average level of FSH, AMH, and E2 in each group during six weeks after UCMSCs transplantation.

Blood was obtained from each rat in all groups at two weeks, four weeks, and six weeks after UCMSCs transplantation. The effects of UCMSCs transplantation on the hormone secretion (FSH, E2, and AMH) of rats were analyzed. Effect indicators were compared among CTX treatment group and UCMSCs transplantation groups for all of these time points. The serum FSH, E2, and AMH levels were all significantly different among these groups (blank control group, CTX control group, and UCMSCs trans groups) (*p* = 0.004, *p* = 0.001, and *p* < 0.0001, resp.). Serum FSH of blank control group maintained a significantly low level (*p* = 0.001), with its E2 and AMH maintaining a significantly high level (*p* < 0.0001), comparing with that in CTX control group. The serum FSH level decreased significantly after UCMSCs transplantation both in tail trans group and in* in situ* trans group comparing with that in CTX control group (*p* = 0.001, *p* = 0.009, resp.) (Figure 5(a)). The serum E2 level has a significant recovery after UCMSCs transplantation for both trans groups comparing with that in CTX control group (*p* < 0.0001) (Figure 5(b)). Our study reported that UCMSCs transplantation has a significant effect on AMH levels in POF rat models for tail trans group comparing with that in CTX control group (*p* = 0.023), but not for* in situ* trans group (*p* > 0.05) (Figure 5(c)). The level of E2 and AMH at different observed time point was significantly different (*p* = 0.002, *p* = 0.002, resp.), while FSH level was not significantly different at different observed time points (*p* > 0.05). The data has been presented in Table 3.

### 3.6. Effect of UCMSCs Transplantation on Follicle Development

**Figure 6:**
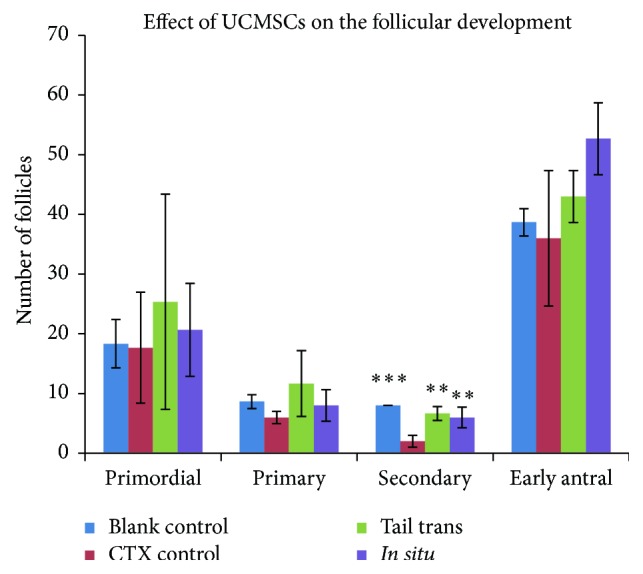
Effect of UCMSCs transplantation on follicular development. Ovarian H&E staining sections were from blank control group, CTX control group, and UCMSCs transplantation groups (by tail intravenously or* in situ*). Follicles were numbered and classified. Data were means ± SD of counts of different stages follicles in three experiments (secondary follicles of UCMSCs treatment groups versus POF control group: *p* < 0.01; primordial, primary, and early antral follicles of UCMSCs treatment groups versus POF control group: *p* > 0.05, *n* = 3). ^*∗∗*^ *p* < 0.01; ^*∗∗∗*^ *p* < 0.001.

Three rats of each group were killed at 6 weeks after UCMSCs transplantation. All the stages of follicles (primordial, primary, secondary, and early antral follicles) were analyzed (Figure 6). The same follicle counting method and statistical comparison method were adopted as those used in Effect of CTX on Follicle Development. Our results indicated a significant increase in secondary follicles in UCMSCs trans groups (both in tail intravenous group and in* in situ* group) as compared to POF group without UCMSCs transplantation (blank control group versus CTX control group, *p* < 0.0001; tail trans group versus CTX control group, *p* = 0.001; and* in situ* trans group versus CTX control group, *p* = 0.003); however, no significant change was observed in the number of primordial follicles, primary follicles, and antral follicles among all the groups (*p* > 0.05). The data has been presented in Table 4.

### 3.7. UCMSCs Transplantation Reduced Ovarian Cells' Apoptosis in POF Rats

**Figure 7:**
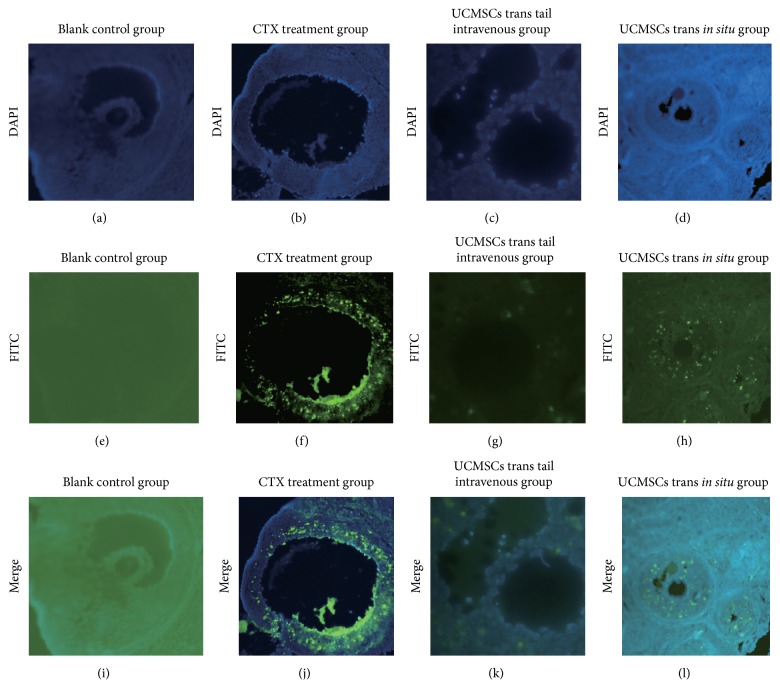
Cell apoptosis assay: UCMSCs transplantation reduced cell apoptosis of ovaries in CTX-induced POF rats. (a, b, c, d) Blue fluorescence indicates cell nucleus stained by DAPI (blank control group, CTX treatment group, tail intravenous trans group, and* in situ* UCMSCs trans group). (e, f, g, h) Green fluorescence (FITC) stained cells illustrating the degree and site of apoptosis of cells in the four groups. (i, j, k, l) merged the two stainings.

Cells apoptosis in ovarian tissues was detected by TUNEL. A number of apoptotic cells were observed in the ovarian tissues of CTX treatment group; ovaries from control group showed mostly healthy follicles with no sign of mounts of cells apoptosis, which was attributed to UCMSCs transplantation. According to our results, after two weeks of UCMSCs transplantation (both* in situ* trans group and tail intravenous trans group), the number of apoptosis cells (FITC-positive cells) in ovary sections was reduced as compared to that in nontrans group (POF group) (Figure 7).

### 3.8. Human UCMSCs Infiltrated into Rat Ovarian Tissue

**Figure 8:**
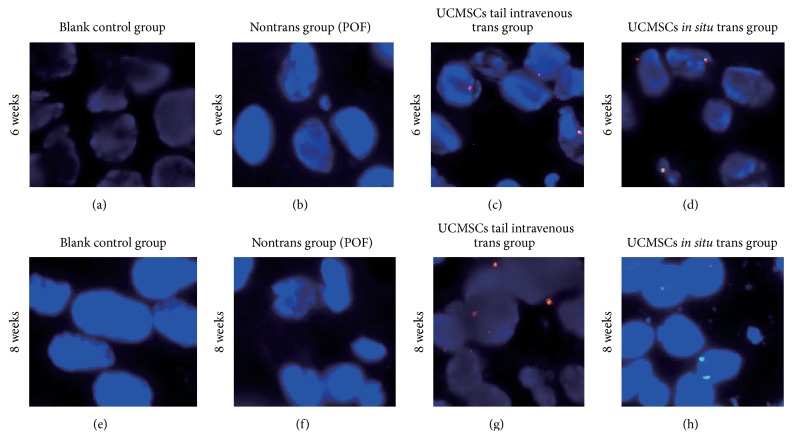
FISH analysis of UCMSCs, tracking of the transplanted human UCMSCs in rats ovarian tissue. (a, b, c, d) Distribution of human UCMSCs in rats ovaries after transplantation for six weeks (blank control group, CTX control group, tail intravenous trans group, and* in situ* trans group). (e, f, g, h) Distribution of human UCMSCs in rats ovaries after transplantation for eight weeks. The location of the UCMSCs was determined using orange (SRY/Y chromosome) and green (CEP/X chromosome) signals. Double-labeled staining (red and green fluorescence) cells were defined as human UCMSCs.

FISH analysis showed the spatial location of transplanted human UCMSCs in ovary tissues. The analysis tracked the fate of the transplanted human UCMSCs at different time points in ovary. Double-labeled staining (red and green fluorescence) cells were defined as human UCMSCs (Figure 8). UCMSCs detected in the ovarian tissues were derived from UCMSCs transplanted groups at six and eight weeks after UCMSCs transplantation. The amount of UCMSCs in the rat ovarian tissue was basically constant, without obvious proliferation during this period, both in tail intravenous trans group and* in situ* trans group. The UCMSCs, however, were not detected in blank control group and POF control group (nontrans group).

## 4. Discussion

The potential of chemotherapy in inducing POF has attracted more concern recently [22, 23]. Since chemotherapy agents could trigger cells into a programmed cell death pathway, continual renewal of ovarian cells makes them more vulnerable to chemotherapy agents. In our study, we observed that CTX administration significantly reduced the ovarian size of POF rats. Ovarian GCs are the most important stroma cells in ovary, which surround oocyte and play a key role in folliculogenesis [24]. Based on its potential of secreting growth factors and providing hormone supplementation, GCs are critical for oocyte growth and survival [25]. The development of follicles always comes along with active GC proliferation and apoptosis and in deciding the fate of follicles in adult human ovaries. Overapoptosis of GCs induced by chemotherapy is the main cause of follicular over-atresia and POF [26]. Our results revealed that chemotherapy severely induced GCs' apoptosis in POF rat models. Further, ovarian pathological analysis presented that CTX administration significantly decreased the number of secondary follicles, while having not much influence on follicles in other stages. Such a phenomenon may be due to the blooming GCs proliferation in this stage. Studies have shown that gonadotropin-releasing hormone analog (GnRHa) may protect the ovary from the effect of chemotherapy by inhibiting its proliferation and follicles growth [27].

Stem cells transplantation is an ideal potential treatment for repairing injured tissue and is also a powerful tool in restoring fertility and pregnancy [28]. Various types of stem cell have been investigated in POF treatment, such as adipose-derived stem cells (ADSCs) [29], human amniotic fluid-derived stem cells (hAFCs) [30], and skin-derived mesenchymal stem cells (SMSCs) [31]. Although an ideal and promising treatment for POF, stem cell transplantation also has some debate going on about its exact mechanism. Some reports supported the fact that transplanted stem cells could be induced to differentiate into ovarian tissue-like cells [32], while others have supported stem cells could help improve the damaged ovarian niches.

There are several advantages for UCMSCs over other MSCs, such as having poor immunogenic properties, which is attributed to their low expression of human leukocyte antigen major histocompatibility complex I (MHC I) and the absence of MHC II molecules [33, 34]. UCMSCs can be easily isolated and expanded* in vitro*. Additionally, they exhibit little ethical issues as compared to other types of stem cells. The cell-surface markers of UCMSCs have been characterized as positive for CD29, CD73, CD90, and CD105 and negative for CD14, CD79, CD34, and CD45, HLA-DR [15, 35]. To date, only limited studies have been conducted determining the role of UCMSCs in POF therapy. Our study systematically proved its efficiency and safety in repairing the damaged rat ovary function induced by chemotherapy.

In our study, the isolated human UCMSCs expressed stem cells markers and exhibited the characteristics of mesenchymal stem cells, which was consistent with previous reports. According to our study, there was a significant reduction in cell apoptosis after the UCMSCs transplantation and improvement in the folliculogenesis in POF rat models. In order to clarify the mechanism of human UCMSCs in repairing the damaged ovarian function in POF rats, it is imperative to trace the fate of the transplanted cells. The UCMSCs, used in our study, were derived from the umbilical cord of new born male, which helped us to detect its location and growth situation in the ovarian tissue by labeling its X and Y chromosome by FISH.

According to FISH results, the transplanted UCMSCs resided in ovarian tissue and survived for at least eight weeks. The cell homing ability of the UCMSCs was based on the expressing mounts of cell adhesion molecules, which aids in the migration of these cells to the target tissues [36]. According to previous reports, UCMSCs could reduce GC apoptosis by affecting its G-protein coupled receptor protein signaling and MAPK pathways, both of which are important for follicle and oocyte growth [37]. UCMSCs could secrete a number of angiogenic growth factors, such as hepatocyte growth factor (HGF), vascular endothelial growth factor (VEGF), placental growth factor (PGF), and TGF-*β*. Many of these factors have been found to have the ability to repair the damaged ovarian function [38]. We speculated that the improved ovarian function in POF rat model was more likely due to the cytokines produced by UCMSCs via paracrine action. However, this speculation needs further investigation.

Endocrine system produces a variety of steroidal hormones, which could regulate follicle growth and survival by paracrine or autocrine mechanism. Disordered hormone secretion is not the only cause but also the outcome of POF pathological process [39]. FSH is produced by adenohypophysis, which exerts its action on the ovary by stimulating follicles growth. FSH receptors (FSHR) are mainly expressed on GCs [40]. Increased level of FSH could accelerate follicles storage depletion; both the cause and the marker diminish ovary reserve [41, 42]. E2 exerts negative feedback on FSH production in the hypothalamus-pituitary glands axis, which is mainly produced by GCs. Overapoptosis of GCs induced by chemotherapeutic agents often results in decreased E2 level. The interrupted negative feedback results in uncontrolled increase in the level of FSH [43]. AMH, which is expressed by GCs of the primary follicles or early antral follicles, acts as an inhibitory growth factor in the ovary during the early stages of folliculogenesis [44]. Usually, the level of AMH is stable during the whole menstrual cycle. Its level strongly correlates with the size of the follicle pool [45].

**Figure 9:**
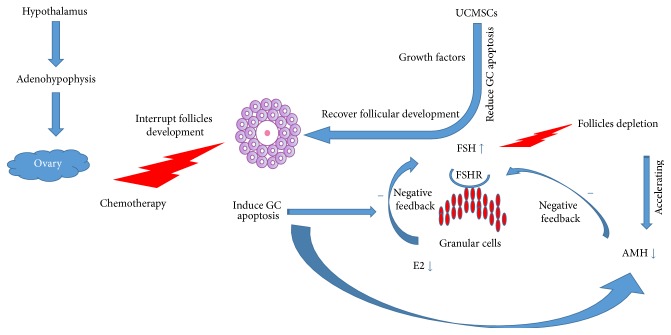
The network of steroidal hormones regulation system during pathological process of POF and UCMSCs therapeutic mechanism. With the hypothalamus regulation, adenohypophysis could synthesize and produce gonadotropins (FSH), which could stimulate follicles growth. GCs of the growth follicles could produce E2, having a feedback negative effect on FSH production, thus reducing follicles excessive growth and avoiding their depletion. AMH could also inhibit FSH production. The negative feedback effect of E2 and AMH on the follicles growth was interrupted by CTX administration. Lack of inhibition of E2 and AMH causes uncontrolled elevating FSH level, which promotes follicles pool exhausted very soon, leading to POF. The transplanted human UCMSCs may secrete kinds of growth factors, which could rescue the overapoptosis of GCs and ultimately improve follicles development and retargeted hormonal balance.

Our study gave a comprehensive report on the disordered steroidal hormone secretion in POF rats induced by CTX administration. Human UCMSCs transplantation improved the disturbed endocrine secretion system to some degree, which is an important mechanism for improving the damaged ovary function. We illustrated the hormone secretion in hypothalamus-adenohypophysis-ovarian axis during the pathological process of POF and the possible therapeutic mechanism of UCMSCs (Figure 9).

## 5. Strengths and Limitations

There are several strengths and limitations of our present study. We monitored the effects of CTX administration on endocrine system function at several CTX posttreatment intervals and concluded that ovarian function declined with time after treatment. Analysis of the change in hormone levels after human UCMSCs transplantation indicated that restoration of the ovarian function indeed takes place to some degree. An ideal therapeutic window for UCMSCs transplantation should be suggested by further study. Additionally, UCMSCs used in our experiment were derived from male baby; Y chromosome was considered as its marker, which could avoid any effects of the cell label on stem cells' survival.

The tumorigenic potential of stem cells therapy has always been an obstacle in its broad application. We traced the transplanted human UCMSCs in rat ovarian tissues at different time points. Although our results revealed that the transplanted human UCMSCs could survive for a long time without obvious proliferation, our experiment did not last long enough to follow the ultimate fate of these cells. Additionally, we could not exclude the possible tumorigenicity potential of these cells. We did not investigate the fertility of the rat model following our current CTX administration protocol and UCMSCs therapy. Further and deeper investigation is required to better understand the exact mechanism of the therapeutic potential of human UCMSCs in POF.

## 6. Conclusion 

In conclusion, our present study systemically studied the efficiency of human UCMSCs in POF rat models, such as human UCMSCs transplantation improving the disturbed endocrine secretion system, reducing the cell apoptosis of ovary, and improving the folliculogenesis. The safety and involved mechanisms of human UCMSCs in POF treatment still need further investigation.

## Supplementary Material

The study design has been listed as Table S1. We established chemotherapy-induced POF rat models according to the protocol listed in the table. UCMSCs transplantation was conducted by tail intravenously and *in situ*.

## Figures and Tables

**Table 1:** Serum levels of FSH, AMH, and E2 determined by ELISA at different CTX posttreatment intervals in three groups (blank control group, saline treatment group, and CTX treatment group). Data were expressed as means ± SD (*n* = 4).

	0 weeks	1 week	2 weeks	3 weeks
FSH (IU/L)				
Blank control	12.559 ± 1.485	12.294 ± 1.096	12.131 ± 0.834	13.294 ± 0.334
Saline control	10.621 ± 0.987	12.303 ± 1.292	13.690 ± 0.941	12.780 ± 1.239
CTX treatment	12.661 ± 0.404	14.401 ± 0.946	15.467 ± 0.782	18.164 ± 1.660
E2 (ng/L)				
Blank control	52.146 ± 12.187	61.213 ± 4.385	53.824 ± 4.080	52.349 ± 10.963
Saline control	52.532 ± 3.097	55.330 ± 4.087	52.601 ± 9.040	51.927 ± 4.936
CTX treatment	49.284 ± 5.980	40.298 ± 7.885	38.095 ± 4.475	35.356 ± 4.567
AMH (pg/mL)				
Blank control	166.288 ± 11.144	152.035 ± 9.665	174.385 ± 14.816	188.845 ± 21.469
Saline control	176.817 ± 15.977	165.875 ± 12.584	161.025 ± 13.914	174.635 ± 18.181
CTX treatment	180.490 ± 13.310	129.340 ± 14.390	153.680 ± 17.830	139.420 ± 7.700

**Table 2:** Counts of different stages follicles were presented as means ± SD (*n* = 3) (primordial, primary, and early antral follicles of CTX treatment group versus blank control group: *p* > 0.05, secondary follicles of CTX treatment group versus blank control group: *p* = 0.004).

	Primordial	Primary	Secondary	Early antral
Blank control	40.7 ± 9.2	14.3 ± 2.5	8.3 ± 1.5	20.3 ± 4.5
CTX treatment	23.0 ± 1.7	15.0 ± 6.2	4.7 ± 1.5	28.3 ± 3.8

*p*	ns	ns	0.004	ns

**Table 3:** Levels of serum hormone analysis and effect of UCMSCs transplantation on the serum hormone secretion. FSH, AMH, and E2 levels were determined by ELISA at two, four, and six weeks after UCMSCs transplantation. CTX control group indicates the nontransplanted POF group. Data were means ± SD (*n* = 4).

	2 weeks later	4 weeks later	6 weeks later
FSH (IU/L)			
Blank control	8.695 ± 0.284	9.802 ± 0.396	8.702 ± 1.384
CTX control	10.864 ± 0.559	11.513 ± 0.643	11.034 ± 0.920
Tail trans	9.763 ± 0.815	9.985 ± 0.501	9.351 ± 0.774
*In situ* trans	11.205 ± 1.438	8.080 ± 1.805	9.493 ± 0.670
E2 (ng/L)			
Blank control	48.629 ± 1.417	54.427 ± 9.108	55.404 ± 4.012
CTX control	40.367 ± 5.342	38.922 ± 8.033	45.696 ± 2.786
Tail trans	53.974 ± 4.045	45.865 ± 3.701	57.248 ± 6.939
*In situ* trans	44.557 ± 2.967	55.594 ± 9.580	55.331 ± 5.060
AMH (pg/mL)			
Blank control	170.242 ± 7.855	142.408 ± 20.819	179.391 ± 10.748
CTX control	113.699 ± 18.022	121.590 ± 5.094	128.758 ± 9.514
Tail trans	139.670 ± 5.843	139.603 ± 19.995	145.132 ± 5.134
*In situ* trans	132.111 ± 12.203	135.545 ± 9.847	153.435 ± 16.090

**Table 4:** Counts of different stages follicles were presented as means ± SD (*n* = 3) (primordial, primary, and early antral follicles of POF group versus UCMSCs transplanted group: *p* > 0.05, secondary follicles of POF group versus UCMSCs transplanted group: *p* < 0.05).

	Primordial	Primary	Secondary	Early antral
Blank control	18.3 ± 4.0	8.7 ± 1.2	8.0 ± 0	38.7 ± 2.3
CTX control	17.7 ± 9.3	6.0 ± 1.0	2.0 ± 1.0	36.0 ± 11.4
Tail trans	25.3 ± 18.0	11.7 ± 5.5	6.7 ± 1.2	43.0 ± 4.4
*In situ* trans	20.7 ± 7.8	8.0 ± 2.6	6.0 ± 1.7	52.7 ± 6.0

*p*	ns	ns	<0.05	ns
