# Supplementary figures and images for: Human Umbilical Cord Mesenchymal Stem Cells Therapy in Cyclophosphamide-Induced Premature Ovarian Failure Rat Model
Source: Biomed Res Int. 2016 Mar 7;2016:2517514. doi: 10.1155/2016/2517514 (PMC4800076; doi:10.1155/2016/2517514)

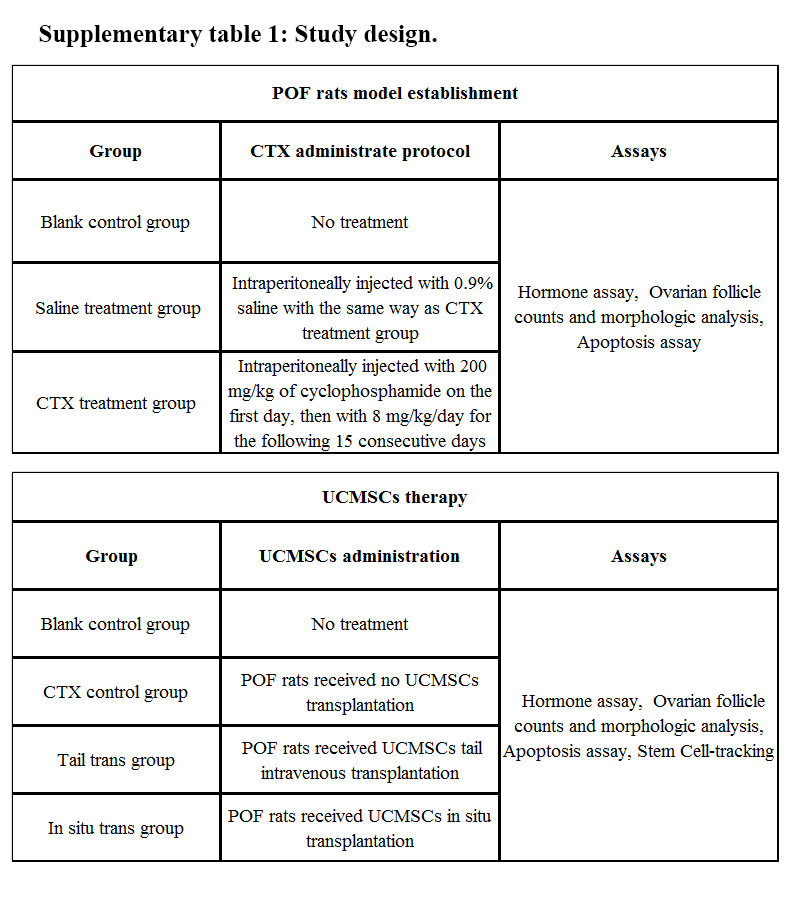

Supplement: Supplementary file 1 — The study design has been listed as Table S1. We established chemotherapy-induced POF rat models according to the protocol listed in the table. UCMSCs transplantation was conducted by tail intravenously and in situ. [file 2517514.f1.tif]
